# The essential role of combined calcium and vitamin D supplementation in the osteoporosis scenario in italy: Expert opinion paper

**DOI:** 10.1007/s11657-024-01451-x

**Published:** 2024-10-22

**Authors:** Stefano Carugo, Fabio Vescini, Andrea Giusti, Giulia Letizia Mauro, Laura Tafaro, Francescaromana Festuccia, Lucia Muraca, Paolo Menè, Maurizio Rossini

**Affiliations:** 1 Department of Clinical Sciences and Community Health, University of Milan, 20122 Milan, Italy; 2 Department of Cardio-Thoracic-Vascular Diseases, Foundation IRCCS Ca’ Granda Ospedale Maggiore Policlinico, Milan, Italy; 3 Endocrinology Unit University Hospital of Udine, Udine, Italy; 4 Division of Internal Medicine, Department of Medicine & Cardiology, “Villa Scassi” Hospital, Genoa, Italy, ASL3, 16132 Genoa, Italy; 5 Department of Precision Medicine in the Medical, Surgical and Critical Care Area (Me.Pre.C.C.), University of Palermo, 90127 Palermo, Italy; 6 Department of Clinical and Molecular Medicine, Sapienza University of Rome, Sant’Andrea Hospital, Rome, Italy; 7 Division of Nephrology, Sant’Andrea University Hospital, Rome, Italy; 8 Department of Primary Care, ASP Catanzaro, 88100 Catanzaro, Italy; 9 Division of Nephrology, Sapienza University of Rome, Sant’Andrea University Hospital, Rome, Italy; 10 Department of Medicine, Rheumatology Unit, University of Verona, Verona, Italy

**Keywords:** Osteoporosis; calcium, Vitamin D, Fragility fracture prevention, Nephrolithiasis risk, Cardiovascular risk

## Abstract

**Summary:**

An Italian multidisciplinary working group discussed the current Italian scenario of osteoporosis management during a meeting and highlighted the essential role of calcium and vitamin D supplementation in the prevention of fragility fractures.

**Purpose:**

This paper aims to review and discuss data on calcium and vitamin D requirements and the role of combined calcium and vitamin D supplementation in the treatment of patients with osteoporosis.

**Methods:**

The discussion of the experts covered literature data on calcium and vitamin D supplementation, gaps in the diagnosis and treatment of osteoporosis, and the role of the primary care physician in identifying and treating patients with osteoporosis. Articles for consideration were identified through PubMed searches using different combinations of pertinent keywords.

**Results:**

The discussion highlighted that insufficient calcium or vitamin D intake increases the risk of fragility fractures. The experts also drew attention to the essential role of calcium and vitamin D supplementation in achieving an anti-fracture effect and supporting the efficacy of anti-osteoporotic agents without increasing nephrolithiasis and cardiovascular risks. In addition, the discussion underlined the role of the primary care physician in the initial clinical approach to patients with osteoporosis.

**Conclusions:**

The experts believe that efficient treatment for patients with osteoporosis should include calcium and vitamin D supplementation to achieve adequate levels that are able to inhibit the parathyroid hormone and bone resorption.

## Introduction

Osteoporosis affects more than 75 million people in the United States, Europe, and Japan and occurs mainly in menopausal women and individuals over 70 years of age due to aging [1]. It is a major public health concern [2], representing the fourth chronic pathology with the greatest impact on disability due to fragility fractures [3]. Fragility fractures profoundly impact patients’ quality of life and have high economic and social burdens [3]. In addition, some fractures are associated with premature mortality, especially those of the hip and spine. Indeed, about 30–38% of deaths after a clinical hip or spine fracture [4–7] can be attributed to the fracture event itself. Although fractures are less frequent in men, the mortality after a hip fracture is higher in this population [8]. In France, Germany, Italy, Spain, the UK, and Sweden, total fragility fractures are expected to increase by 23%, from 2.7 million in 2017 to 3.3 million in 2030. The annual fracture-related cost was €37.5 billion in 2017 and is expected to increase by 27% in 2030 [3]. Italy is the second European country with the highest rate of osteoporosis, and the number of fractures is expected to increase by 20% in 2034 to a total of 700,000 fractures [9–11]. The number of deaths caused by fragility fractures is estimated at 105 per 100,000 inhabitants in the population over 50 years of age; it is equivalent to, and in some cases exceeds, the number of deaths caused either by lung cancer, diabetes, or chronic lower airway diseases [11]. Osteoporosis is diagnosed by measuring bone mineral density (BMD) through a dual-energy X-ray absorptiometry (DXA) scan or when the occurrence of non-traumatic hip or vertebral fractures is observed [12]. DXA screening is recommended to predict fracture risk in women older than 65 years or those who present risk factors [13, 14]. DXA screening is also recommended in men over 70 years; conversely, it is advised for men aged 50–69 or < 50 years old in the presence of fragility fractures and one or more major risk factors [15]. Osteoporosis treatment aims to prevent fragility fractures and stabilize or increase BMD [16]; it includes non-pharmacological management, such as adequate calcium and vitamin D intake, lifestyle changes [17–19], and pharmacological therapy [20].

The role of calcium and vitamin D in the pathophysiology of osteoporosis is relevant. The rate of bone resorption also depends on calcemia, which is finely regulated by the calcium-parathyroid hormone (PTH)-vitamin D axis [21]. In the case of hypocalcemia, PTH is secreted and increases calcium mobilization from the bones. PTH stimulates vitamin D activation, increasing intestinal absorption and renal calcium reabsorption [22, 23], thus making it available for bone mineralization [24]. Vitamin D inhibits PTH secretion, reducing the effect of bone resorption [25]. Furthermore, it has been observed that vitamin D supplementation is effective in suppressing PTH and bone resorption when combined with calcium [26, 27]. Therefore, optimal vitamin D and calcium levels should be achieved to ensure the effectiveness of anti-osteoporosis treatment [28–30].

This article aims to review and discuss the anti-fracture effect of combined calcium and vitamin D supplementation and its essential role in the management of patients with osteoporosis. It will also highlight the role of the primary care physician (PCP) in prescribing this supplementation in Italy, along with a presentation and discussion of the skeletal and possible non-skeletal benefits and hypothetical risks of this supplementation.

## Methodology

An Italian multidisciplinary working group composed of endocrinologists, cardiologists, nephrologists, PCPs, geriatrics, and rheumatologists discussed the current scenario of osteoporosis management in Italy at a meeting. The discussion covered gaps in the diagnosis and treatment of osteoporosis, guidelines for calcium and vitamin D supplementation, and the possible benefits and risks of these supplementations. The authors also conducted a narrative review of the literature to provide an overview of the topic. Articles for consideration were identified through several PubMed searches using different combinations of pertinent keywords (“fragility fractures”; “fragility fractures in Italy”; “osteoporosis in men”; “osteoporosis” AND “calcium supplementation”; “osteoporosis” AND “vitamin D supplementation”; “calcium and vitamin D supplementation”; “calcium homeostasis”; “osteoporosis” AND “fracture risk assessment tools”; “fragility fractures” AND “secondary prevention”). Subsequently, the authors selected the most significant studies for this paper.

## Results

### Anti-fracture effect of vitamin D and calcium supplementation

Data in the literature support the anti-fracture effect of combined calcium and vitamin D supplementation, while in some studies, vitamin D supplementation alone does not appear to have a significant effect [31, 32]. LeBoff et al. recruited healthy men over 50 years and healthy women over 55 years old, regardless of their vitamin D status, low bone mass, or osteoporosis. Although the paper had some selection biases, it showed that vitamin D alone cannot reduce the risk of fractures in the healthy adult population [32]. In a meta-analysis, Weaver et al. showed that calcium and vitamin D supplementation in adults led to a 15% reduction in fracture risk [33]. In another meta-analysis, Yao et al. showed that vitamin D supplementation alone did not significantly reduce the risk of fractures of any type and hip fractures. On the other hand, in the group of patients treated with combined calcium and vitamin D supplementation, for every 10.0 ng/mL increase in vitamin D level, there were 7% fewer fractures and 20% fewer hip fractures. They also showed that the daily calcium supplementation combined with vitamin D for approximately 6 years resulted in a significant reduction in the risk of any fracture by 6% and hip fracture by 16% [31]. Liu et al. showed that combined calcium and vitamin D supplementation significantly reduced hip fractures by 14% in postmenopausal women with osteoporosis [34]. Supplementation of these nutrients is also supported by studies showing that sufficient calcium intake, alongside sufficient vitamin D levels, reduces PTH levels [21] and increases BMD [35].

#### Expert opinion

During the discussion, the authors emphasized that calcium supplementation in combination with vitamin D has a clear anti-fracture action. They stressed that the plasma calcium level does not indicate an adequate calcium intake, as the calcium-PTH-vitamin D axis sustains normal plasma calcium levels by sequestering it from bones. Therefore, even in protracted and severe deficits in calcium intake and reduced BMD, PTH-induced bone resorption ensures normal plasma calcium levels. In this context, the shared opinion holds that lowering serum PTH levels is critical in the treatment of osteoporosis. Importantly, as previously reported, this combination is able to reduce PTH levels [21] and, therefore, is effective in preventing bone resorption. The authors stated that insufficient calcium intake correlates with an increased risk of fractures and that its correction reduces the risk of fracture events [36].

In conclusion, the authors endorsed the prescription of combined calcium and vitamin D supplementation given its significant anti-fracture effect, which is associated with decreased PTH levels and bone resorption.

### Calcium and vitamin D supplementation: The italian guidelines and the current scenario

**Fig. 1:**
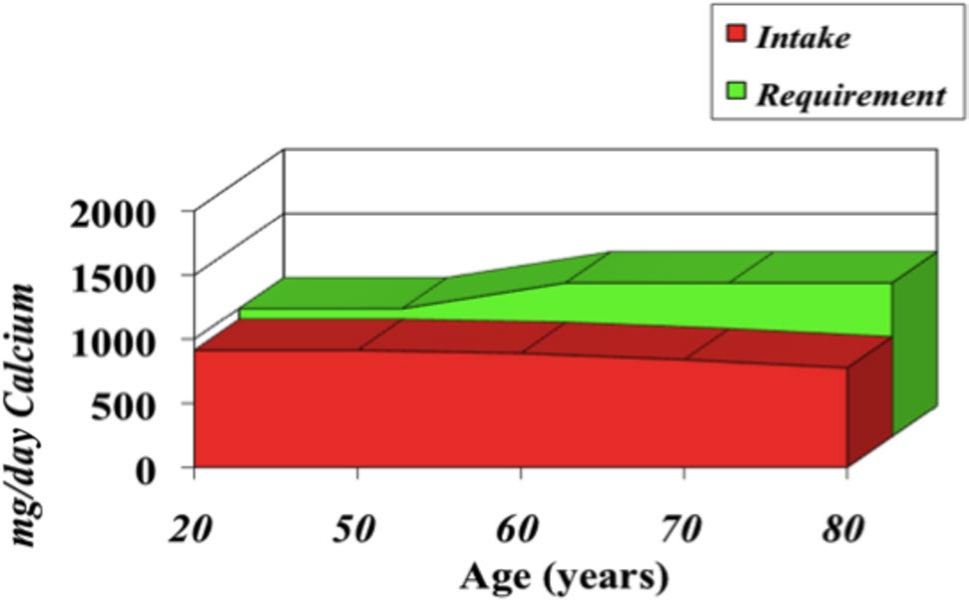
Calcium intake versus calcium requirements by age. Data taken from [13, 94]

According to the Italian Society for Osteoporosis, Mineral Metabolism, and Bone Diseases (SIOMMMS), the level of serum 25-hydroxy vitamin D should be maintained at 20–50 ng/mL in the general population and at 30–50 ng/mL in the population at risk of fragility fractures or on treatment with bone-modifying agents, recommending that plasma 25-hydroxy vitamin D be monitored over time in the latter categories. Vitamin D intake in patients with hypovitaminosis D and candidates for bone-active agents for osteoporosis should be 800–2000 IU per day. SIOMMMS claims that the nutritional approach in Italy is insufficient to achieve the requirement, and supplementation is very often necessary. Adequate calcium intake of 800–1000 mg/day should always be ensured in the general population [37]. Specifically, 1200 mg/day of calcium intake is suggested in postmenopausal women and men older than 65 years [13]. Although awareness of the need for proper calcium and vitamin D intake has been promoted [38], a Canadian study showed that calcium and vitamin D insufficiency was widespread in 2004 and was still extremely diffused in 2015. This study showed that 90% of over-50-year-old patients with heart disease and/or osteoporosis did not have adequate vitamin D intake in 2004 and continued to lack it in 2015. In the osteoporotic population who reported being on supplementation, only 5% did not reach adequate vitamin D concentrations. On the other hand, the situation regarding calcium intake worsened from 2004 to 2015, as dietary calcium intake in patients with osteoporosis further decreased from 648 to 580 mg, a reduction of 600 mg from the 1200 mg recommended by the guidelines [39]. These data emphasize the need for adequate calcium and vitamin D supplementation, especially in the older population. Indeed, calcium requirements increase with age; however, with advancing age, calcium intake is reduced (Fig. 1).

There is evidence that insufficient calcium intake correlates with increased fractures; similarly, increasing calcium intake decreases the risk of fractures [36].

However, it has been shown that anti-osteoporotic therapy should be established only if adequate calcium and vitamin D levels are reached [29, 40]. In Italy through the publication of *Note*, the Italian Medicines Agency (Agenzia Italiana del Farmaco, AIFA) defines the therapeutic indications for which a certain medicinal product can be charged to the National Health Service. In this regard, the Italian Medicines Agency (Agenzia Italiana del Farmaco, AIFA) recommended in *Nota 79* the use of vitamin D supplementation for patients who display fragility fractures, patients at risk of fragility fractures, or patients initiating anti-osteoporotic therapy [41]. Nevertheless, Italian real-life data showed that low calcium intake predominates among patients at risk of decreased BMD or fractures, as only one-fifth have sufficient calcium intake [42].

Furthermore, to reduce inappropriate prescriptions of vitamin D, in 2019, AIFA published *Nota 96*, which limits the reimbursement of vitamin D supplements (cholecalciferol and calcifediol) for both the prevention and treatment indication of vitamin D deficiency [43]. However, this led to a significant decrease in vitamin D prescriptions even in patients who needed to increase their vitamin D levels because at risk of fractures and/or on bone resorption drugs. Although 20% of patients on drug treatment for osteoporosis were not taking calcium and vitamin D supplements before *Nota 96*, this rate has currently doubled, with a lack of supplementation in 40% of this population [44].

#### Expert opinion

During the expert discussion, it was pointed out that the older population has been neglected, and awareness of the need for calcium and vitamin D supplementation, especially in this population and not only during the fall and winter months, is crucial. It should be noted that AIFA *Nota 96* does not cover conditions at risk of vitamin D deficiency, such as those related to the inability to produce adequate amounts of vitamin D despite exposure to sunlight, or advanced age [45]. Also, *Nota 96* conditions in which there is reduced sun exposure, due to working indoors, using the veil or burka, and due to conditions contraindicating exposure to UVB rays, such as lupus erythematosus or photosensitivity due to diverse conditions [46], are not taken into account. The authors stressed that adequate calcium and vitamin D intake in the Italian population is hindered by the scarcity of these nutrients in foods that are not fortified in calcium and vitamin D. Indeed, the authors stated that supplementation is fundamental to compensate for the deficiency due to diet.

Looking at the European scenario, only Finland, the UK, Iceland, Sweden, and Ireland consistently fortify food with calcium and vitamin D. Fortification is also permitted but rarely adopted in Germany, Denmark, Austria, and Switzerland [47]. The lack of fortified foods could explain the global prevalence of inadequate calcium and vitamin D dietary intakes, except for Northern Europe [48–50].

The authors stated that the absence of fortified food is one of the reasons for the already widespread calcium and vitamin D insufficiency in the Italian population and that supplementation is fundamental. In addition, the experts drew attention to the efficacy of vitamin D only in patients with an intake of 1200 mg of calcium through the diet. They reported that the proportion of patients with sufficient calcium intake is very low, which is the reason behind the ineffectiveness of vitamin D supplementation alone. Therefore, vitamin D supplementation should be combined with adequate dietary calcium intake or the administration of calcium supplements. To better determine the calcium requirements for each patient, the experts emphasized the need to consider dietary calcium intake, the rates of renal absorption and calcium excretion, and the consumption of foods that inhibit its absorption. This way, it would be possible to estimate the amount of calcium that should be supplemented to reach adequate concentrations. The authors recommended that calcium and vitamin D should be adequately supplemented. Patients should be followed up by detecting PTH levels, 24-h urinary calcium excretion, and the levels of 25-hydroxy vitamin D. The authors reported that the SIOMMMS guidelines indicate that the serum vitamin D value cut-off is 30 ng/mL of 25-hydroxy vitamin D [37] below which supplementation is necessary. About the calcium supplements, the dose of 600 mg seems to be optimal in terms of absorption, as calcium absorption rises rapidly when increasing the dose from 0 to 500 mg while only slightly increments when the dose is increased above 500 mg [51, 52]. This is due to the ability of the gastrointestinal to efficiently absorb calcium at 500 mg at a maximum ingestion dose at a time. Therefore, eventually, higher doses of calcium tablet supplements, rarely necessary in clinical practice, should be fractioned and taken several times daily [38, 53].

The experts agreed that combined supplementation of 600 mg calcium and 2000 IU vitamin D daily is fundamental in patients with osteoporosis who exhibit inadequate dietary calcium intake and serum 25-hydroxy vitamin D levels. They stated that daily supplementation of these nutrients over bolus supplementation is preferable because it can mimic a physiological condition. In their opinion, daily supplementation exerts all the effects of vitamin D throughout the day, both at the skeletal and non-skeletal levels, with improved outcomes. They reported that cholecalciferol supplementation is better documented in terms of anti-fracture efficacy and more physiological than calcifediol. Indeed, besides the cholecalciferol activation, which is finely regulated by the calcium-PTH-vitamin D axis, cholecalciferol has its own actions on body functions [54]. Based on the literature, they also pointed out that calcium carbonate is a highly bioavailable and rapidly absorbed formulation [55], in particular, when taken with a meal and not in a fasting state [56]. Furthermore, since calcium must be taken daily, the use of a combined calcium and vitamin D pill is a simple approach to dual supplementation and is generally, more well accepted by patients [57] who also appreciate the greater suitability of orodispersible formulations, especially in the older population [58]. The authors affirmed that many PCPs do not prescribe vitamin D supplementation due to misinterpretation of AIFA *Nota 96*, resulting in the thwarting of anti-osteoporotic treatment. Consequently, they stressed the importance of disseminating information about supplementation and the appropriate dosages within the healthcare system and the general public.

In conclusion, the authors endorsed the essential role of combined calcium and vitamin D supplementation for patients at risk of fragility fractures.

### The possible risks of calcium and vitamin D supplementation: Myths or facts?

#### Cardiovascular risk of vitamin D supplementation?

According to the reported role of vitamin D deficiency in the increased mortality and incidence of cardiovascular diseases [59], some clinical studies have shown that vitamin D supplementation is not a risk factor for the cardiovascular system [60, 61]. Indeed, the VITAL clinical trial showed that vitamin D and omega-3 supplementation had no cardiovascular effect but exhibited a promising signal for reducing total cancer mortality. In addition, an increased risk of hypercalcemia, kidney stones, and gastrointestinal symptoms was not observed [60]. In the VIDA trial, after a median of 3.3 years of vitamin D supplementation, despite an increase in serum 25-hydroxy-vitamin D concentrations by more than 20 ng/mL compared with placebo, there was no reduction in cardiovascular diseases, death, myocardial infarction, stroke, heart failure, or venous thromboembolism [62].

#### Cardiovascular risk of calcium supplementation?

Calcium supplementation has previously been described as increasing the frequency of cardiovascular events [63]. Although vascular calcification has been hypothesized as the mechanism through which calcium increases cardiovascular disease events [60], this hypothesis was raised based on data from subjects with renal insufficiency who are at increased risk of vascular calcification [64–66]. However, subsequent studies have shown no correlation between calcium supplementation and increased cardiovascular risk. Michaëlsson et al. showed that dietary calcium intake of 600–1400 mg/day was not associated with higher death rates from all causes, cardiovascular disease, ischemic heart disease, and stroke [67]. Furthermore, a meta-analysis showed no consistent dose–response relationship between calcium intake and cardiovascular mortality [68]. Higher dietary calcium intake has been associated with reduced risks of cardiovascular events, mortality [36], and reduced risk of pre-eclampsia among pregnant women on a low-calcium diet [69]. Besides, there is a consensus in the literature that calcium supplementation combined with vitamin D does not appear to be harmful to the cardiovascular system [70]. Indeed, a previous study also demonstrated the safety of long-term calcium and vitamin D supplementation; no differences in coronary artery calcium scores were observed in women who supplemented 1000 mg of calcium and 400 IU of vitamin D daily for 7 years compared with the placebo group [71]. Based on the literature, the National Osteoporosis Foundation and the American Society for Preventive Cardiology stated that moderate-quality evidence indicates that calcium, whether or not combined with vitamin D intake from food or supplements, does not alter the risk of cardiovascular mortality or all-cause mortality in healthy adults. Therefore, they suggested that calcium intake up to 2000–2500 mg/day should be considered safe from a cardiovascular standpoint. They also stressed that discontinuing calcium supplementation for safety reasons is unmotivated and may be harmful to bone health when sufficient dietary intake is not achieved [72].

#### Nephrolithiasis risk of calcium and vitamin D supplementation?

Malihi et al. showed that an average of 3.3 years of monthly supplementation with 100,000 IU of vitamin D3 did not influence the incidence rate of kidney stones or hypercalcemia [73]. Regarding calcium, Taylor et al. showed that higher dietary calcium from non-dairy or dairy sources is independently associated with a lower kidney stone risk [74]. In another study, Malihi et al. showed that the combined calcium and vitamin D supplementation did not increase the risk of kidney stones [75]. Oral calcium and vitamin D intake after 1 year did not affect urinary calcium excretion rate and kidney stone formation in postmenopausal women. No significant differences were found between 24-h urinary and blood calcium levels before and after 1 year of treatment with calcium (1000 mg/day) and vitamin D (400 IU/day). After 1 year, an ultrasound examination confirmed asymptomatic lithiasis in one of 53 patients (1.9%) [76]. Hence, in a recent study, Messa et al. stated that the nephrolithiasis risk should also be assessed alongside the analysis of the effectiveness of calcium and vitamin D supplementation on bone health parameters. They suggested that this risk should be analyzed through the changes in urinary calcium excretion, the appearance of hypercalcemia or de novo occurrences, or relapses of nephrolithiasis events [77]. Conversely, Wallace et al. showed that in the Women’s Health Initiative clinical trial, renal stone risk was 16% higher in those taking calcium and vitamin D supplementation for 5 years compared with placebo. However, this may have been due to the fact that personal calcium supplements were allowed and the mean baseline calcium intake in the participants was 1100 mg/day. Moreover, some participants in the study had a personal history of stone disease, in violation of the protocol, and were not excluded in the intention-to-treat analysis [78].

#### Expert opinion

The authors agreed that there is neither an increased risk of nephrolithiasis nor evidence of increased cardiovascular risk in patients supplementing calcium and vitamin D. However, they stated that not well-documented information on the cardiovascular risk has raised fears and is causing widespread calcium and vitamin D deficiency. They reported that the number of patients with cardiovascular risk demonstrating calcium and vitamin D deficiency is steadily increasing. In this scenario, the authors affirmed that supplementing 600 mg of calcium combined with 2000 IU of vitamin D daily is safe and is not associated with increased renal and cardiovascular risk.

In conclusion, the authors highlighted that calcium and vitamin D supplementation is safe and highly necessary to prevent fragility fractures.

### Secondary prevention and treatment gaps in europe and italy: The role of the PCP

Secondary prevention through pharmacologic treatment is critical to reduce the rate of subsequent fragility fractures, improve health outcomes, and reduce healthcare costs [79]; it should be adopted along with calcium and vitamin D supplementation in older people and post-menopausal women who sustained a low-trauma fracture [80]. In France, Germany, Italy, Spain, the UK, and Sweden, the number of patients with osteoporosis not treated with anti-osteoporotic agents increased by 17% between 2010 and 2017. The treatment gap was 73% for women and 63% for men, while in Italy, it was 48% for men and 73% for women [3]. In all member countries of the European Union, the UK, and Switzerland, the treatment gap in 2019 was 71%, meaning that 15 million women eligible for osteoporosis therapy did not receive the treatment [9]. The International Osteoporosis Foundation audit 2020 showed that osteoporosis is a priority in Italy, and the availability of DXA equipment per million population, as well as guidelines for osteoporosis diagnosis and treatment, is adequate [9]. Despite this, Italy ranks last in the management of osteoporosis [3]. The management of patients with osteoporosis who suffered fractures has been defined as a “Bermuda Triangle” composed of orthopedists, PCPs, and osteoporosis experts in which the patient disappears [81]. To avoid this scenario, it is strongly recommended that the patient with fragility fractures is managed by systematic and coordinated models of care, which have been established in numerous hospital settings and are known as fracture liaison services (FLSs). FLSs rely on multidisciplinary services that combine hospital-based teams (e.g., emergency and orthopedic), primary care, bone specialists, nurse coordinators, physical therapists, and dietitians [80, 82]. To provide secondary preventive care, recognizing fragility fractures and initiating an investigation into their causes or treatment is crucial. The investigation should include BMD, spinal imaging, risk factors, and identification of possible causes of low bone mass. The role of the PCP is essential in identifying patients at high risk of fragility fractures, and algorithms that combine clinical risk factors with BMD, such as the Fracture Risk Assessment Tool (FRAX®) [83], FRAX-derived tool (DeFRA) [84], and Fracture Health Search (FRA-HS), have been developed for this purpose. In Italy, these fracture risk assessment tools are available to PCPs and could accelerate the request for more diagnostic studies. In addition, these tools could help identify patients in whom calcium and vitamin D should be started, in combination or not, with anti-osteoporosis therapies [85, 86]. After the patient has been discharged from the hospital, communication between the specialist and the PCP is crucial for the long-term management of osteoporosis [87]. Indeed, in a study carried out in the United States, it has been shown that the number of patients with fragility fractures who are treated with anti-osteoporotic drugs increases when bone specialists actively participate and efficiently communicate with the PCP [88–90]. It is important to highlight the need for long-term care of osteoporosis, as many patients do not maintain adherence to anti-osteoporosis treatment. FLSs with a more intensive approach to secondary fracture prevention have proven to be efficient and cost-effective in improving treatment initiation rates; they are also associated with higher adherence to treatment and, consequently, lower refracture risk, reduced mortality, and increased BMD [91–93].

#### Expert opinion

The authors pointed out that risk assessment tools for PCPs are widely available in Italy, particularly DeFRA and FRA-HS [3], which play an essential role in identifying patients with osteoporosis at risk of fragility fractures. They also stated that once identified by the PCP, the patient should be referred to a specialist to establish the most appropriate treatment. Nevertheless, the authors sustained that the PCP should play an essential role in prescribing calcium and vitamin D supplementation. Furthermore, it is known that there are patients at risk of fractures despite being treated with anti-osteoporotic drugs because they do not reach adequate calcium and vitamin D values. In this scenario, the PCP holds a crucial informational role through their trust relationship and can raise awareness of the importance of calcium and vitamin D supplementation in preventing fragility fracture and making anti-osteoporosis treatment effective. The PCP can help patients understand that supplementation is a therapy to which patients must adhere to ensure sufficient calcium and vitamin D intake capable of reducing PTH. Moreover, the PCP is responsible for prescribing vitamin D supplementation according to the concept of appropriateness, even for those patients at risk of hypovitaminosis D who are not covered by AIFA *Nota 96* [37]. Since the PCP cannot prescribe second-tier drugs for the treatment of osteoporosis, the authors emphasized the need to educate bone specialists on the crucial role of prescribing anti-osteoporosis treatments alongside calcium and vitamin D supplementation. Indeed, the collaboration between bone specialists and PCPs is pivotal in patient management.

The PCP should prescribe calcium and vitamin D supplementation if not already prescribed by other specialists and is responsible for the follow-up of patients. Additionally, a Diagnostic Therapeutic Assistance Pathway (PDTA) is needed in all regions to understand how to recover patients. Attention must be paid to the compliance of the patient who does not consider osteoporosis a pathology but a consequence of aging. Therefore, awareness campaigns would be useful to make patients consider osteoporosis as a disease that has the same relevance as hypertension. In cases where it is not known whether the patient has hypovitaminosis D and low calcium intake, it is crucial to take into account risk factors, such as celiac disease and malabsorption syndrome. The authors drew attention to the scarce perception of hypovitaminosis D and the need for a questionnaire to assess calcium and vitamin D intake.

In conclusion, the authors highlighted the essential role of the PCP in identifying patients at risk of fragility fractures and prescribing calcium and vitamin D supplementation as a preventive anti-osteoporotic measure or in combination with anti-osteoporotic agents.

## Conclusions

In order to prevent fragility fractures and to ensure the effectiveness of anti-osteoporotic drugs, sufficient calcium and vitamin D intake, either through diet or supplementation, is pivotal. It is also important to note that despite higher calcium and vitamin D requirements, intake is lower in the older population. Nevertheless, the Italian scenario is characterized by widespread insufficient calcium and vitamin D intake not only in older individuals; this situation has been worsening in recent years as the prescription of supplementation has been discouraged. Therefore, raising awareness of the importance of calcium and vitamin D supplementation in treating osteoporosis is important. In this setting, the PCP plays a central role in the diagnosis and treatment of osteoporosis and in the prescription of calcium and vitamin D supplementation, which contributes to the prevention of fragility fractures and is critical to the success of anti-osteoporotic therapy. Furthermore, it is pivotal to inform the healthcare system and the general public about the recent findings showing that calcium and vitamin D supplementation does not lead to increased renal and cardiovascular risk. The key aspects that emerged from the discussion of the experts are summarized in Box 1.

**Box 1** Key highlights of expert opinions.

**Table:** 

• Insufficient calcium or vitamin D intake is correlated with increased fractures
• Supplementation is fundamental to achieve adequate calcium and vitamin D levels, which are often not attainable through diet alone and can decrease PTH
• Vitamin D has an anti-fracture effect only when adequate calcium intakes are achieved
• Calcium supplementation in combination with vitamin D has a clear anti-fracture action
• Calcium supplementation in combination with vitamin D supports the efficacy of anti-osteoporotic agents
• The dose of 600 mg calcium and 2000 IU vitamin D is often essential to obtain adequate serum levels of calcium and 25-hydroxy vitamin D, as recommended by the guidelines
• Correction of calcium or vitamin D deficiency does not increase the risk of nephrolithiasis
• Calcium intake, combined with vitamin D, does not increase cardiovascular risk
• The primary care physician plays a key role in the initial clinical approach of the patient with osteoporosis
